# Exploring the role of financial empowerment in mitigating the gender differentials in subjective and objective health outcomes among the older population in India

**DOI:** 10.1371/journal.pone.0280887

**Published:** 2023-01-23

**Authors:** Shreya Banerjee, Pallabi Gogoi

**Affiliations:** Centre for the Study of Regional Development, Jawaharlal Nehru University, New Delhi, India; Indian Institute of Technology Roorkee, INDIA

## Abstract

**Background:**

Despite the progress in achieving gender equality to a certain extent, women are found to be more susceptible to health disadvantages compared to men in the older ages. However, research in the Indian context has mainly remained restricted to subjective health that heavily depends on the individual’s perception, which may affect the validity of results. This study addresses this gap by complementing the investigation of the gender differentials in self-reported health outcomes (mobility and functional limitations) with that of objectively measured health status (hand-grip strength and static balance) among the older population of India. Besides, there is a dearth of literature that considers financial empowerment in explaining the gender differentials in health. Women’s ability to participate in household decision-making, especially for important matters like major purchases, including property, indicates their empowerment status. Furthermore, the ability to extend financial support can be considered an important ‘non-altruistic’ driver for kins to care for older adults, indirectly affecting their health and well-being. Thus, the present paper explores the influence of financial empowerment on gender differentials in poor health outcomes.

**Methods:**

Using the Longitudinal Aging Study in India, Wave-1 (2017–18), six logistic regression models have been specified to capture the adjusted association between gender and poor health outcomes. The first three models successively control for the demographic and social support factors; socioeconomic factors and pre-existing health conditions; and financial empowerment indicators. The last three models investigate the interactions between gender and marital status, living arrangement and involvement in financial decisions, respectively.

**Results:**

The findings reveal that women tend to be more perceptive about their physical discomfort than men and reported a higher prevalence of poor subjective health. In terms of objectively measured health status, older men had a higher prevalence of low hand-grip strength but a lower prevalence of poor balance. Gender demonstrated a strong, adjusted association with poor health outcomes among older adults. However, the magnitude of gender difference either shrunk considerably or became statistically insignificant for all the poor health outcomes after controlling the effect of indicators of financial empowerment. Further, the interaction between gender and involvement in financial matters demonstrated a stronger effect for men in reversing poor subjective health.

**Conclusion:**

The study reinforced the positive effect of financial empowerment in mitigating gender disparity in health among older adults.

## Introduction

Population aging is unquestionably one of the most transformative demographic phenomena of the 21^st^ century. Increasing longevity, declining mortality, and the progression of large-sized population cohorts to the older ages are crucial factors in explaining the rising share of the older population across the world. The World Health Organization (WHO) has estimated that the proportion of the world’s older population aged over 60 years will be doubled between 2015 and 2050 [1]. Similar to the world trend, India is also expected to experience a rise in the share of older population from 8.6 percent in 2011 to 20 percent in 2050 [2]. However, the older population is highly vulnerable to functional disability and chronic diseases [3]. The increased vulnerability manifested in old age, in tandem with poor perceived health, thus, brings the importance of providing special attention to the ageing population for health and well-being.

Subjective health, in terms of self-assessed health, is one of the widely used measures of health outcomes. The self-assessed health is multidimensional in nature and includes physical, functional, and well-being aspects of the individuals. As self-reported health (SRH) is also indirectly associated with objective health, literature suggests that SRH was preferred more than any other individual disease condition in health analysis [4–7]. However, self-reported health status tends to differ depending on an individual’s understanding and responses which may affect the validity of results [8, 9]. This requires adjustments for the differences, validation and, if possible, the adaptation of some supporting objective measures.

Despite the progress in achieving gender equality to a certain extent [10], women are found to be more susceptible to health disadvantages as compared to men in the older ages. The universal fact that women live longer than men spurred some discussions on their subjective and objective health status [11, 12]. A few studies support the argument that the poorer health of women compared to men is based on higher levels of morbidity [13–15] in terms of health-related quality of life and chronic diseases. The health survival paradox of women, i.e., living longer than men with higher morbidity, can be explained by biological factors [16] as well as socioeconomic position [17]. However, research in developing countries, such as in the Indian context has not focused much on the health status of the older population in respect of both subjective and objective health status.

The health survival paradox of women is found to be insignificant in most developed countries. Several studies found minimal or diminishing or no gender differentials in health among older adults in the case of both subjective and objective health [18–21]. Educational attainment, labour force participation, and leisure activities are considered the most important factors which explain the existence or non-existence of gender differentials [22]. Since the educational attainment is almost similar for both men and women in most developed countries, the gender gap in health either became negligible in those countries or shrank with time.

In contrast, evidence from developing countries suggests the prevalence of gender differences in both subjective and objective health measures, thereby providing more evidence for the health survival paradox of women [23–25]. Kieny et al. (2021) investigated the gender differences in subjective well-being among the older population in LMICs and found that women were disadvantaged both in evaluative and emotional well-being [25]. This suggests the existence of gender differentials in socioeconomic status and health. Anand et al. (2020) found education, marital status, and employment status as the most important factors in explaining the gender differentials in frailty and functional limitation [24]. Moreover, these factors restrict the mobility and social connectivity of women, making them more exposed to health disadvantages [26, 27].

According to United Nations (UN), India falls under the ageing or greying country as its population aged 60 and above reached 8.6% in 2011 [2]. The challenges resulting from the changing pattern of India’s population need special attention. It carries various social, economic, and health implications such as reduction in the labour force, expenditure on social security, including pension, etc. [28, 29]. Despite modernization, India’s patriarchal norms made women more disadvantageous in all spheres of life [30]. Furthermore, India has been experiencing a profound gender differential with respect to health status and health care utilization [4, 31]. Older women are particularly at higher risk of neglect, isolation, poverty, and dependency. Because of low literacy, societal norms, gender relations, and lack of social networking, older women are unable to avail the benefits of various social security programmes [32]. The plight of being women and old at the same time might confirm the double jeopardy hypothesis, which means “combined negative effects of occupying two stigmatized statuses” can be “greater than occupying either status alone” [33].

Though younger women are becoming more educated, urbanized, and progressing in their professional careers with time, most of the older women are yet far from modernization and socio-economically backward than their male counterparts [34]. Women being more sensitive to their physical discomfort than men, are expected to overreport those discomfort [35]. On the contrary, men usually consider their health age-appropriate and, consequently, might underreport their self-rated health [36]. Because of the lack of large-scale data availability in developing countries, literature on gender inequalities in subjective and objective health is scarce. Similarly, the pattern of gender differences in health among older adults is complex and context-specific, which needs special attention.

Only the conventional biological and socioeconomic variables might not be sufficient for measuring the gender gap in both objective and subjective health among older adults [19, 37]. Women tend to accumulate a lesser share of household wealth than their male counterparts and have a lesser influence on household economic and other important decision-making [38, 39] which might contribute to gender inequality in health. While most of the studies have assessed the demographic and socioeconomic spheres in analyzing the gender difference in health status among the older population, there is a dearth of literature that takes into account the financial empowerment in explaining the gender difference in both subjective as well as the objective health status of the older population. Women’s ability to participate in household decision-making, especially for important matters like major purchases, including property, indicates their autonomy or empowerment level [39]. Furthermore, the ability to extend financial support can be considered an important ‘non-altruistic’ driver for kins to care for older adults, indirectly affecting their health and well-being [40].

With this background, the paper aims to analyse the gender differentials in health outcomes, if any, in respect of both subjective as well as objective health status among older adults. The study also investigates the existence of any relationship between subjective and objective health status. Moreover, this study explores whether financial empowerment influences gender differentials in both subjective and objective health status among older adults. The study considers financial empowerment in terms of intra-household decision-making and financial support, which would significantly contribute to the existing literature on determinants of gender differences in health among older adults.

### Theoretical background

The present paper largely follows the framework of social determinants of health to explore the factors responsible for both subjective and objective health status of older adults [41–43]. Social determinants of health refer to both specific features of and pathways by which societal conditions affect health that potentially can be altered by informed action [41]. Social determinants imply the conditions where people are born, live and work within the systems and institutions affecting their quality of life [44].

**Fig 1:**
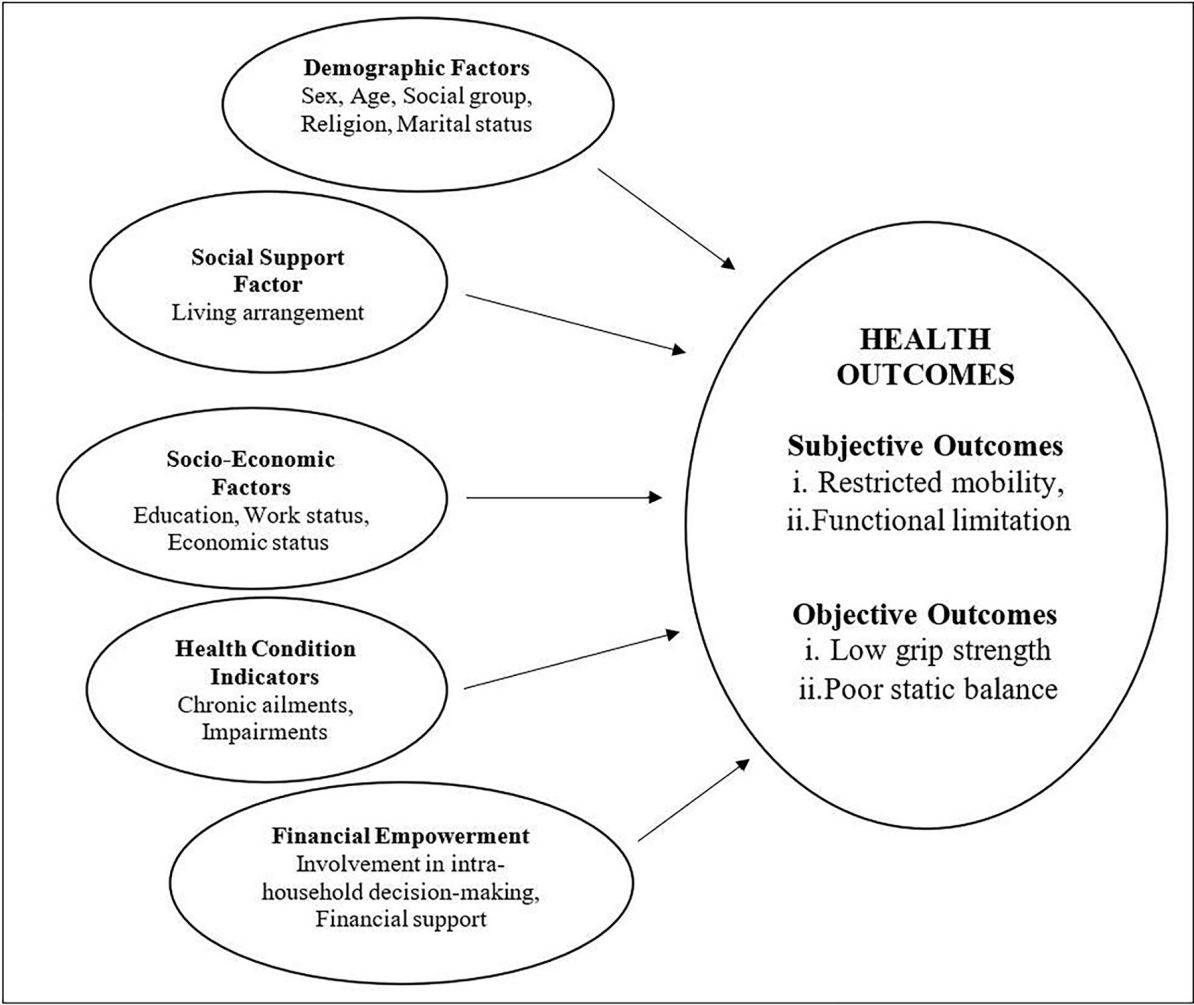
The conceptual framework of determinants of health outcomes.

The Model (Fig 1) demonstrates how various factors like demographic, social support, socioeconomic, health condition, and financial empowerment indicators determine the health status of older adults. Demographic variables like sex, age, marital status, social groups, and religion are important predictors which tend to directly or indirectly affect the health status of older persons. Most demographic variables are considered to have low mutability as they are irreversible [45]. Sex is a biological factor associated with differential risk levels; however, it has some socioeconomic underpinnings with respect to resource allocation, social position, empowerment, etc. [46]. While the genetic or biological aspect of sex lacks mutability, its socioeconomic aspect can be understood to have a certain degree of mutability.

Age may negatively affect subjective health status but with a differential effect across gender [40]. With the increase in age, people are more likely to experience several complex health conditions. Many older people experience a significant decline in physical and mental capacities and a growing risk of disease. Marital status may influence health status in several ways. The increase in social networks which results from marital relationships may help people to access health-related information and services and encourage them to resort to a healthy lifestyle. In addition, it also provides increased access to resources to avail quality health-care services [47]. Social groups (caste and tribes) and religion are also vital factors in determining individual outcomes in all spheres of life [48]. Most of the time, certain caste affiliation appears as a hindrance in receiving supply-side benefits. Caste and religion not only reflect the social status of an individual but also the constraints they face to access the social resources [42, 49].

Educational attainment, work status, economic status, and place of residence are considered under the domain of socioeconomic factors. Educational attainment positively affects the health outcome through increase in knowledge and awareness. It also has mediating effects through enhanced income and employment opportunities [50]. Differences in educational attainment may lead to disparities in healthy ageing. Work status has been considered an important co-variate of mortality and morbidity. Being employed may help improve health status [51, 52] by providing means to health care in terms of regular income as well as health insurance etc. On the other hand, being unemployed may impact not only physical health but also mental health. Economic status influences lifestyle choices necessary for healthy ageing and provides resources to deal with poor health [53, 54]. Place of residence refers to the regional variation in terms of stages of development. In rural areas, poor economic resources, low opportunities, lack of quality health care services, etc., are the factors that affect the overall quality of life. However, urban areas with poor environmental conditions and other lifestyle-related factors may also impact health status directly or indirectly [55, 56].

Living arrangement can be considered a social support factor determining social security in the absence of pension or other social security schemes [40]. Living with children or family may provide financial security on one hand and psychosocial support on the other hand. Pre-existing health conditions like chronic ailments and impairments may also impact both subjective and measured health outcomes of older persons. The prevalence of chronic ailments, which increases with age, is known to be negatively associated with health status [57]. Financial empowerment or autonomy, which can be considered one of the most important factors providing older people the ability to take greater control of their health and well-being [58], may impact a person’s health status to a greater extent.

Finally, both subjective and objective health status are taken into account as health outcomes. The study considers restricted mobility and functional limitation as subjective health outcomes and low grip strength and poor static balance as objective health outcomes.

## Material and methods

### Data source

The analysis has been done using the wave-1 data of the Longitudinal Aging Study in India (LASI) conducted during 2017–18 [59]. The LASI is a nationally representative large-scale sample survey that adopted a multistage stratified area probability cluster sampling design and interviewed 72250 older adults aged 45 and above (including their spouses irrespective of age) across all states and union territories of India covering 42949 households. The survey collected data on the burden of disease, functional health, health-care utilisation, and the socioeconomic well-being of older adults. In addition, the LASI also included several internationally validated biomarker tests to assess the participants’ physiological, performance-based, anthropometric and dried blood spot based molecular measurements. In case the selected respondent had severe cognitive or physical impairment, a proxy interview was done, in which case, biomarker assessments were not conducted. For the present analysis, only the respondents aged 60 years or above (31464; 15098 males and 16366 females) were considered. Due to the non-availability of biomarker data pertaining to objective health measures, the sample sizes for the outcomes: hand-grip strength and static balance were 28 095 (13 560 males,14 535 females) and 26 836 (12 994 males, 13 843 females), respectively.

### Ethics statement

The Longitudinal Aging Study in India (LASI) Wave-1 (2017–18) was granted ethical approval by the Indian Council of Medical Research (ICMR). Participants were provided with information brochures containing details of the purpose of the survey, confidentiality, safety of the biomarker assessment tests, etc. to ensure informed consent. Informed consent (signed/oral) was obtained from age-eligible participants both for the interviews as well as the biomarker assessments. More details regarding the ethics protocols can be found in the LASI (Wave-1) India report [60]. Separate ethical clearance was not obtained for this study as the authors did not conduct a primary data collection and only utilised unit-level anonymised data available in the public domain.

### Outcome variables

Since older adults have demonstrated a tendency to report a more positive perception of health divergent from their objective health status in later life [61] we have chosen two measures each of subjective and objective health outcomes. The two subjective health outcomes were derived from a set of related items. Therefore, the scale reliability coefficient (Cronbach’s alpha) was calculated to check the internal consistency. The Cronbach’s alpha (*α*) can be calculated as follows [62]:

$$\alpha =\frac{N*c}{v+(N-1)c}$$ (1)

N is the number of items,

*c* is the average inter-item covariance among the items, and

*v* is the average variance

Cronbach’s alpha values range between 0 and 1. The general rule of thumb is that a value of greater than or equal to 0.70 but less than 0.80 indicates acceptable, 0.80 or above, but below 0.90 denotes good, and 0.90 or above indicates excellent internal consistency or scale reliability.

#### i. Subjective health outcomes

- ***Restricted mobility***: Respondents were asked if they had any difficulty in performing a total of nine activities related to mobility. The Cronbach’s alpha measured 0.87, suggesting good scale reliability. For the present analysis, if the respondent indicated a ‘yes’ to having difficulties (that had lasted for more than three months) in five or more activities, they were considered to have a restricted mobility (yes = 1), otherwise not (no = 0).
- ***Functional Limitation***: The LASI assessed difficulty faced in performing a total of thirteen Activities of Daily Living (ADL) due to a physical, mental, emotional or memory problem. The Cronbach’s alpha measured 0.91, suggesting an excellent internal consistency. If the respondent indicated a ‘yes’ to having difficulties (that had lasted for more than three months) in five or more activities, they were considered to have a functional limitation (yes = 1), otherwise not (no = 0).

#### ii. Objective health outcomes

- ***Low grip strength***: This is a measure of upper body muscle strength. The associated health risks with low hand grip strength include frailty, falls, and functional limitations. The LASI measured grip strength in kilograms using Smedley’s Hand Dynamometer, and two readings of grip strength each for both hands were recorded. In this study, we have considered the average of the two measurements of the participant’s dominant hand. In cases where the measurement of the dominant hand was not performed due to reported surgery, injury, etc., the measurements of the non-dominant hand were considered. It has been demonstrated that the muscle mass of an average Indian is lower compared to an average Caucasian [63] hence, applying western cut-offs may yield misleading results [61]. Therefore, for the present analysis, in accordance to the standards suggested by the Asian Working Group for Sarcopenia, low muscle strength has been defined as hand-grip strength <28 kg for men and <18 kg for women [64]. Thus, a binary variable for low grip strength was created (yes = 1, if below cut-off; otherwise, no = 0).
- ***Poor static balance***: Poor static balance is associated with disability, risk of falls and neurological conditions. In the LASI, the mid-level balance test (semi-tandem) was conducted first, followed by a more (or less) challenging position if the respondent succeeded (or failed) to perform the semi-tandem test. Those who could successfully hold the semi-tandem position for 10 seconds were subsequently asked to maintain their balance in the full-tandem position (for 60 and 30 seconds for individuals aged <70 and ≥70 years, respectively). However, if the participant failed the semi-tandem test, the side-by-side stance was asked to be performed for 10 seconds. In the present analysis, a dichotomous variable for poor static balance was created (yes = 1, if failed to perform semi-tandem or full tandem; no = 0 otherwise).

### Predictor variable

Based on the Social Determinants of Health framework, five broad domains of co-variates have been identified that may induce inequalities in subjective and objective health outcomes. These domains pertain to demographic factors, social support factors, socioeconomic factors, health conditions, and financial empowerment indicators.

**Demographic factors** include sex (male and female); age (younger olds (60–69 years), old-olds (70–79 years) and oldest olds (80 years and above)), age and age-squared have been used as continuous variables in the multivariate regression models; marital status (currently married (including those in live-in relationships) and others (including never married/ divorced/ separated/ widowed)); social groups (Scheduled Castes (SC), Scheduled Tribes (ST), Other Backward Classes (OBC) and others); and religion (Hindu, Muslim, and others (comprising all other minority religious groups like Christians, Sikhs, etc.)). **Social support** is measured by living arrangement (living alone and not alone).

The **socioeconomic variables** include level of education (illiterate, upto primary, secondary, higher secondary or above); work status (never worked, currently not working/ unpaid work, currently working and being paid); economic status (represented by monthly per-capita consumption expenditure-based wealth quintiles); place of residence (urban and rural). **Health condition indicators** relate to the number of prevailing chronic ailments (none, only one, two or more); and impairments (none, only one, two or more).

Financial empowerment has been measured in varied ways by different studies based on the feasibility of the dataset being analysed as well as the scope of the study. For instance, Roy and Chaudhuri (2008) [40] used property ownership and economic independence as proxies of financial empowerment while analysing the National Sample Survey, India. Ali et al. (2021) [65] and Postmus et al. (2013) [66] used an adaptation of the family empowerment scale (FES) [67], consisting of six items, as an instrument to measure financial empowerment in their studies. Moonzwe Davis et al. (2014) [68] constructed a multidimensional Women’s Empowerment Scale with ‘control over decisions and finances’ as one of the major components encompassing women’s participation in the household in matters of saving money or purchasing goods. In this study, we have identified three variables of participation in intra-household decision-making and financial support that can be considered excellent proxies of the level of financial empowerment. These variables are defined as follows:

■ Role in decisions regarding ‘buying and selling of property’–categorised as no role, decides alone, contributes jointly with other household members.
■ Involvement in ‘payment of bills and settling of financial matters’- categorised as yes or no.
■ Financial support–questions were asked about giving as well as receiving monetary support (amounting to more than Rs 1000 in the past one-year recall period) to and from family or friends. In this analysis, the responses were categorised into four combinations: received but not given, neither received nor given, received and given, not received but given.

### Statistical analyses

Descriptive statistics have been used to examine the means and percentages of co-variates by gender. Bivariate percentage distribution has been calculated to estimate the gender differentials in the prevalence of poor health outcomes (subjective as well as objective) by predictor variables. The results were tested for statistical significance by using Pearson’s Chi-squared test for homogeneity or independence [69]:

$$\chi^{2}=\sum \frac{{\left(O_{i}-E_{i}\right)}^{2}}{E_{i}}$$ (2)

x^2^ is the chi squared value,

O_i_ are the observed values, and

E_i_ are the expected values

Spearman’s rank-order correlation coefficients were calculated to examine the relationships between the subjective and objective health outcomes [70]:

$$\rho =1-\frac{6\sum d_{i}^{2}}{n(n^{2}-1)}$$ (3)

ρ is the Spearman’s rank correlation coefficient,

d_i_ is the difference between the ranks of each observation, and

n represents the total number of observations

Six logistic regression models have been specified to capture the adjusted association between gender and poor health outcomes: restricted mobility, functional limitation, low grip strength, and poor static balance. For each health outcome, the first model (Model 1) controls for the demographic and social support factors. The successive two models (Models 2 and 3) control for socioeconomic factors and pre-existing health conditions; and financial empowerment indicators, respectively. The last three models (Models 4, 5 and 6) investigate the interactions between gender and marital status, living arrangement, and involvement in financial matters, respectively, controlling the co-variates considered in Model 3.

The binary logistic models used to examine the association between health outcomes and the independent variables can be expressed by the following equation [71]:

$$P_{i}=Pr(Y=1|X=x_{i})=\frac{\exp\left({\beta_{o}+x}_{i}\beta_{n}\right)}{1+exp(\beta_{o}+x_{i}\beta_{n})}+u$$ (4)

P _i_ is the probability of poor health outcome (restricted mobility, functional limitation, low grip strength, and poor static balance), x _i_ is the vector of co-variates of i^th^ individual, the coefficients β _n_ are parameters to be estimated, and *u* is the error term. The interaction models include a predictor variable formed by multiplying two ordinary predictors, for example, gender and marital status (x _1_ * x _2_) and β _n’_ is the coefficient of the interaction term, expressed as follows.


$$P_{i}=Pr(Y=1|X=x_{i})=\frac{\exp\left(\beta_{o}+x_{i}\beta_{n}+(x_{1}*x_{2})\beta_{n\prime }\right)}{1+exp(\beta_{o}+x_{i}\beta_{n}+(x_{1}*x_{2})\beta_{n\prime })}+u$$ (5)

The odds ratios (OR) are computed as (P _i_ /1- P _i_).

Sample weights, provided in the LASI (2017–18) dataset, have been applied in the analyses to account for selection probabilities and adjust for non-response in order to accurately reflect the structure of Indian population. All the statistical analyses have been done using the software STATA (version 16).

## Results

### Gender differentials in key demographic, social support, socioeconomic, financial empowerment, and health indicators

The results of the descriptive analysis, presented in Table 1, revealed that significant gender differences exist in select co-variates among older adults. The prevalence of widowhood is higher among older women in comparison to older men. With respect to living arrangement, an important indicator of social support, a higher proportion of women reported to be living all by themselves. Furthermore, older women reported significantly higher levels of illiteracy than their male counterparts. An average older man has attended 3 additional years of schooling than an average older woman. The proportion of older adults who have never engaged in paid work for at least three months in their lifetime is higher among women. Older women also reported a higher rate of suffering from multi-morbidities than older men. The vulnerabilities of the older women are aggravated by their considerably lower levels of role and involvement in intra-household decisions related to property and finances. A higher share of older women has received (but not given) monetary support from family/ friends, while a higher share of older men has given (but not received) such support. These gender differences in key demographic and Socioeconomic Status (SES) indicators clearly serve to the disadvantage of older women as the potential for worse health outcomes due to widowhood, solitary living, poor SES, and lack of financial empowerment has been well documented in previous studies [40, 47, 72].

**Table 1:** Gender-wise distribution of the sample by select background characteristics.

Background Characteristics		Total		Male		Female		Male-female difference	p value
		Frequency^a^	% (or mean)	Frequency^a^	% (or mean)	Frequency^a^	% (or mean)		
**Age group**	Younger olds (60–69 years)	18974	58.51	8961	57.82	10013	59.13	-1.31	<0.001
	Older Olds (70–79 years)	9101	30.20	4545	31.14	4556	29.35	1.79	
	Oldest olds (80 years and above)	3389	11.29	1592	11.04	1797	11.52	-0.48	
**Mean age**			69.17		69.26		69.09	0.17	<0.001
**Marital Status**	Currently Married	20090	62.09	12506	81.72	7584	44.36	37.36	<0.001
	Widowed	10719	36.20	2293	16.49	8426	53.99	-37.5	
	Others	655	1.71	299	1.79	356	1.64	0.15	
**Social Group**	SC	5140	18.91	2448	18.78	2692	19.02	-0.24	0.260
	ST	5173	8.12	2436	7.72	2737	8.48	-0.76	
	OBC	11886	45.23	5781	45.86	6105	44.66	1.2	
	Others	9265	27.74	4433	27.63	4832	27.84	-0.21	
**Religion**	Muslim	3731	11.28	1804	11.72	1927	10.88	0.84	0.482
	Hindu	23037	82.22	11078	82.04	11959	82.39	-0.35	
	Others	4695	6.50	2216	6.25	2479	6.73	-0.48	
**Living Arrangement**	Alone	1622	5.68	365	2.52	1257	8.53	-6.01	<0.001
	With children and spouse/ others	21883	68.25	10456	67.59	11427	68.85	-1.26	
	With spouse and/or others	7959	26.07	4277	29.89	3682	22.61	7.28	
**Education**	Illiterate	17691	58.79	5827	41.01	11864	74.86	-33.85	<0.001
	Upto primary	6758	20.35	4131	26.91	2627	14.42	12.49	
	Secondary	4614	13.89	3307	20.50	1307	7.92	12.58	
	Higher secondary or above	2401	6.96	1833	11.58	568	2.80	8.78	
**Mean years of Schooling**			3.27		4.85		1.83	3.02	<0.001
**Work Status**	Never worked	8776	26.41	755	3.81	8021	46.83	-43.02	<0.001
	Currently not working/ unpaid work	13856	44.50	8300	54.60	5556	35.38	19.22	
	Currently working (paid)	8824	29.09	6039	41.60	2785	17.79	23.81	
**Economic Status**	Poorest	6484	21.70	3035	20.83	3449	22.49	-1.66	0.015
	Poorer	6477	21.71	3068	21.32	3409	22.06	-0.74	
	Middle	6416	20.95	3064	21.60	3352	20.35	1.25	
	Richer	6170	19.19	2990	19.22	3180	19.16	0.06	
	Richest	5917	16.45	2941	17.02	2976	15.93	1.09	
**Place of Residence**	Rural	20725	70.55	10077	72.05	10648	69.18	2.87	0.002
	Urban	10739	29.45	5021	27.95	5718	30.82	-2.87	
**Chronic disease**	None	11525	37.14	5787	38.58	5738	35.86	2.72	<0.001
	Only one	9491	30.73	4524	30.87	4967	30.61	0.26	
	Two or more	10342	32.13	4720	30.55	5622	33.53	-2.98	
**Mean no. of chronic ailments**			1.16		1.10		1.21	-0.12	<0.001
**Impairment**	None	28387	89.55	13552	89.45	14835	89.63	-0.18	0.036
	Only one	1810	6.42	958	6.88	852	6.01	0.87	
	Two or more	1126	4.03	505	3.66	621	4.36	-0.7	
**Mean no. of impairments**			0.16		0.16		0.17	-0.01	0.036
**Role in property related decisions**	No role	5074	18.96	1382	10.35	3692	26.65	-16.3	<0.001
	Decides alone	2920	10.60	1973	16.14	947	5.66	10.48	
	Decides jointly	23014	70.44	11489	73.51	11525	67.69	5.82	
**Involvement in payment of bills/ settling of financial matters**	No	20422	68.27	6961	48.99	13461	85.50	-36.51	<0.001
	Yes	10587	31.73	7885	51.01	2702	14.50	36.51	
**Financial support**	Received and given	610	1.83	332	2.17	278	1.52	0.65	<0.001
	Received but not given	3929	13.38	1592	11.87	2337	14.73	-2.86	
	Not received but given	1419	4.09	945	5.83	474	2.53	3.3	
	Neither received nor given	25001	80.70	11955	80.13	13046	81.22	-1.09	
**TOTAL**		31464	100.00	15098	47.45	16366	52.55	-5.1	

^a^ unweighted sample sizes

Source: Authors’ own calculations from Longitudinal Aging Study in India (LASI), Main Wave I, (2017–18)

### Gender differences in the prevalence of poor subjective and objective health outcomes

Tables 2 and 3 present the gender differentials in the prevalence of poor subjective and objective health outcomes, by select co-variates. The sex-wise prevalence rates of each of the poor health outcomes have been presented in S1 and S2 Appendices. At the national level, 39.7% and 20.6% of older adults reported restricted mobility and functional limitations, respectively, both of which represent subjective health outcomes. However, a considerably higher proportion of older adults were found to have low grip strength (70.2%) and poor static balance (28.1%), when direct health examinations were conducted. The prevalence of restricted mobility, functional limitation, and poor static balance was higher among older women by 13.3, 11.4, and 11.4 percentage points, respectively. However, the rate of poor grip strength was higher among the older men by 4.4 percentage points. By and large, older persons who were the oldest old, widowed, socially disadvantaged, Muslim, living alone, illiterate, currently not working, poorest, rural residents, suffering from multi-morbidities or multiple impairments, having no role in property-related decisions or involvement in other financial matters and in receipt of financial support, reported the highest prevalence rates of poor health outcomes. The gender differences disfavored the older women consistently across all co-variates in the case of both the subjective health outcomes and poor static balance.

**Table 2:** Gender-wise prevalence of poor health outcomes.

	Male	Female	Total
**Restricted Mobility (%)**	32.65	45.99	39.69
**Functional Limitation (%)**	14.58	25.98	20.6
**Low Grip Strength (%)**	72.45	68.06	70.15
**Poor Static Balance (%)**	22.15	33.55	28.08

Note: all p-values for chi-squared test statistic were below 0.001

Source: Authors’ own calculations from Longitudinal Aging Study in India (LASI), Main Wave I, (2017–18)

**Table 3:** Gender differentials in the prevalence of poor health outcomes (subjective and objective) by background characteristics.

Background Characteristics		Male-Female Difference			
		Restricted Mobility	Functional Limitation	Low Grip Strength	Poor Static Balance
**Age group**	Younger olds (60–69 years)	-12.55†	-9.66†	5.63†	-12.19†
	Older Olds (70–79 years)	-16.7	-14.09	2.12	-11.72
	Oldest olds (80 years and above)	-9.32	-13.21	-0.21	-8.63
**Marital Status**	Currently Married	-8.19†	-5.2†	8.57†	-6.89†
	Widowed	-11.45	-11.05	7.19	-8.99
	Others	-1.13	-0.67	17.32	-7.95
**Social Group**	SC	-12.66†	-11.68†	6†	-7.19†
	ST	-8.91	-11.15	4.46	-10.08
	OBC	-11.59	-11.11	4.72	-13.13
	Others	-18.02	-11.81	2.72	-12.24
**Religion**	Muslim	-15.8†	-13.05†	3.13†	-17.54†
	Hindu	-13.33	-11.76	4.37	-10.78
	Others	-9.57	-4.33	6.79	-8.55
**Living Arrangement**	Alone	-9.37†	-1.79†	5.25†	-15.31**
	With children and spouse/ others	-15.02	-13.36	4.21	-11.98
	With spouse and/or others	-7.54	-7.11	6.32	-9.01
**Education**	Illiterate	-8.99†	-8.9†	9.95†	-9.31
	Upto primary	-11.12	-4.63	6.52	-11.87
	secondary	-15.59	-10.94	7.27	-21.38
	Higher secondary or above	-17.29	0.43	1.73	-6.61
**Work Status**	Never worked	-6.28†	-0.62†	8.07†	-8.61†
	Currently not working/ unpaid work	-10.9	-12.87	3.89	-7.1
	Currently working (paid)	-11.21	-6.17	3.74	-7.95
**Economic Status**	Poorest	-13.11	-12.57†	6.84†	-7.05†
	Poorer	-11.5	-9.23	7.2	-9.85
	Middle	-13.08	-10.97	0.86	-7.99
	Richer	-12.15	-14.53	5.7	-18.94
	Richest	-17.67	-8.99	0.82	-15.24
**Place of Residence**	Rural	-13.29	-11.58†	4.95**	-10.22†
	Urban	-13.86	-11.57	2.69	-14.07
**Chronic disease**	None	-12.22†	-10.99†	3.15*	-10.15†
	Only one	-12.59	-10.85	3.75	-8.19
	Two or more	-13.32	-11.43	6.44	-15.3
**Impairment**	None	-14.42†	-11.35†	4.26†	-11.38†
	only one	-0.47	-7.92	6.36	-15.12
	two or more	-7.95	-16.5	2.8	-8.02
**Role in property-related decisions**	No role	-3.55†	-7.19†	6.67†	-15.83†
	Decide alone	-14.54	-10.44	1.95	-11.16
	Decides jointly	-12.27	-7.77	6.11	-8.39
**Involvement in payment of bills/ settling of financial matters**	No	-4.2†	-4.55†	10.94†	-7.02†
	Yes	-11.26	-5.86	4.04	-8.59
**Financial support**	Received and given	-13.66†	-18.18†	8**	-1.27
	Received but not given	-12.41	-9.99	7.46	-7.5
	Not received but given	-10.89	-11.58	-2.24	-16.19
	Neither received nor given	-13.35	-10.96	4.18	-11.87
**TOTAL**		-13.34†	-11.4†	4.39†	-11.4†

Note

† *p* < 0.001

*** *p* < 0.01

** *p* < 0.05 and

* *p* < 0.1

Source: Authors’ own calculations from Longitudinal Aging Study in India (LASI), Main Wave I, (2017–18)

### Correlations between subjective and objective health outcomes

Table 4 presents the correlation coefficients between subjective and objective health outcomes among older adults. In the case of both older men and women, there is a weak or very weak, albeit statistically significant, correlation between the subjective and objective health outcomes. Restricted mobility showed a negative correlation with grip strength and a positive correlation with poor balance. Functional limitation was negatively correlated with grip strength and positively correlated with poor balance. The weak correlations between the self-reported and measured health status revealed that both subjective and objective health measures provide unique insights into the evaluation of an older adult’s health status. The findings align with earlier studies that have found a weak or insignificant correlation between subjective and objective health measures [61, 73–75]. While sole reliance on self-reported health may fail to provide the actual health status of an individual, a wide array of literature has found evidence that subjective health is a more important predictor of mortality and morbidity in comparison to objective measures [76, 77].

**Table 4:** Spearman’s correlations between subjective and objective health outcomes.

	Restricted Mobility	Functional Limitation	Grip Strength	Poor Balance
**Restricted Mobility**	-	0.55^†^	-0.23^†^	0.19^†^
**Functional Limitation**	0.57^†^	-	-0.23^†^	0.16^†^
**Grip Strength**	-0.27^†^	-0.26^†^	-	-0.24^†^
**Poor Balance**	0.19^†^	0.18^†^	-0.22^†^	-

Note

† *p* < 0.001

Correlations for females are above the diagonal and correlations for males are below the diagonal

Source: Authors’ own calculations from Longitudinal Aging Study in India (LASI), Main Wave I, (2017–18)

### Determinants of inequalities in prevalence of poor health outcomes

Table 5 presents the unadjusted association between gender and poor health outcomes. When the health outcomes were regressed on gender, significant gender differences were exhibited with older women demonstrating higher odds of poorer health outcomes (in case of restricted mobility, functional limitation and poor balance) and lower odds of low grip strength.

**Table 5:** Unadjusted association between gender and poor health outcomes.

Dependent Variable Yes = 1, No = 0		Restricted Mobility	Functional Limitation	Low Grip Strength	Poor Balance
Predictor		Crude Odds Ratios			
**Sex**	Male®				
	Female	1.76†	2.06†	0.81†	1.77†

Note: **®**Reference category

† *p* < 0.001

Source: Authors’ own calculations from Longitudinal Aging Study in India (LASI), Main Wave I, (2017–18)

Table 6 presents the adjusted effect of gender on each of the subjective and objective health measures computed through the logistic regression models, controlling for successive vectors of demographic and social support variables; socioeconomic factors and pre-existing health conditions; and empowerment indicators in three different models (models 1–3). When additional controls for demographic and social support (model 1); and socioeconomic factors and health conditions (model 2) were accounted for, the magnitude of the gender differences in health outcomes contracted but persisted and remained significant nonetheless, disfavouring older women in all but prevalence of low grip strength. With the controlling of the effect of indicators of financial empowerment (model 3), the magnitude of gender difference further shrunk but remained statistically significant only for restricted mobility and low grip strength. The gender differences in functional limitation and poor balance were no longer significant upon controlling for the effect of financial empowerment.

**Table 6:** Determinants of poor health outcomes (subjective and objective) among the older population in India.

Dependent Variable Yes = 1, No = 0		RESTRICTED MOBILITY			FUNCTIONAL LIMITATION			LOW GRIP STRENGTH			POOR STATIC BALANCE		
Predictors		MODEL 1	MODEL 2	MODEL 3	MODEL 1	MODEL 2	MODEL 3	MODEL 1	MODEL 2	MODEL 3	MODEL 1	MODEL 2	MODEL 3
		Odds Ratio			Odds Ratio			Odds Ratio			Odds Ratio		
**Sex**	Male®												
	Female	1.73†	1.36†	1.15**	1.96†	1.39†	1.12	0.79†	0.62†	0.56†	1.76†	1.51†	1.37
**Age**	Age	1.11**	1.04	1.04	1.08	0.99	1.00	1.09	1.1	1.11	1.02	0.97	0.97
	Age squared	1.00	1.00	1.00	1.00	1.00	1.00	1.00	1.00	1.00	1.00	1.00*	1.00*
**Marital Status**	Others®												
	Currently Married	0.87**	0.88**	0.9*	0.72†	0.75***	0.79***	0.92	0.96	0.99	0.79***	0.81***	0.85**
**Social Group**	Non- SC/ST®												
	SC/ST	0.95	0.95	0.95	1.19**	1.07	1.08	1.27†	1.14***	1.14**	0.73†	0.81***	0.81***
**Religion**	Muslim®												
	Hindu	0.82***	0.9	0.86**	0.91	1.04	0.97	0.99	1.11	1.12	1.05	1.07	1.07
	Others	0.78***	0.79	0.75***	0.7***	0.78*	0.75**	0.94	1.1	1.11	1.63†	1.56†	1.56†
**Living Arrangement**	Others®												
	Living alone	1.1	1.15	1.32**	0.76***	0.79***	0.83	1.27**	1.3**	1.35**	0.84	0.88	0.87
**Education**	Not literate®												
	upto primary		0.86**	0.87**		0.6†	0.64†		0.76†	0.77†		0.95	0.97
	Secondary		0.66†	0.68***		0.56**	0.63**		0.6†	0.6†		0.97	0.99
	higher secondary		0.55†	0.61†		0.38†	0.48***		0.45†	0.47†		0.69***	0.73**
**Work status**	unpaid work/ not working®												
	Paid work		0.55†	0.62†		0.37†	0.45†		0.78†	0.82†		0.58†	0.62†
**Economic Status**	Poorest®												
	Poorer		0.9	0.93		0.75***	0.78***		0.96	0.97		1.09	1.11
	Middle		0.87**	0.88*		0.71†	0.73†		0.91	0.92		1.21**	1.24***
	Richer		0.8***	0.83**		0.75**	0.75**		0.87	0.91		1.23**	1.27**
	Richest		0.88	0.92		0.66†	0.7***		0.87*	0.89		1.28***	1.34***
**Residence**	Rural®												
	Urban		0.77†	0.81***		0.7†	0.72†		1.1	1.14*		1.14*	1.16**
**Chronic ailment**	None®												
	only one		1.55†	1.55†		1.28***	1.29***		0.97	0.96		1.05	1.05
	two or more		2.81†	2.87†		2.17†	2.17†		0.96	0.96		1.4†	1.38†
**Impairment**	None®												
	only one		1.66†	1.72†		2.09†	2.12†		1.33**	1.29*		1.57†	1.55†
	two or more		3.06†	3.08†		3.74†	3.65†		1.23*	1.23		1.34**	1.35**
**Property related decisions**	No role®												
	Decides alone			0.8**			0.52†			0.87			0.88
	Jointly			0.99			0.61†			0.87			0.77***
**Involvement in financial matters**	No®												
	Yes			0.57†			0.44†			0.74†			0.76†
**Financial support**	Received but not given®												
	Neither received nor given			0.96			0.84*			0.93			1.01
	Received and given			1.04			1.08			1.25			0.87
	Not received but given			0.79**			0.66***			0.9			0.83
**Model Specifications**	Number of observations	31,333	31,328	30,938	31,294	31,289	30,900	28,095	28,092	27,888	26,836	26,834	26,642
	Wald statistic	527.13	1212.3	1462.16	848.81	1377.11	1561.57	583.06	684.75	734.26	586.51	729.44	710.2
	Prob > chi2	0.000	0.000	0.000	0.000	0.000	0.000	0.000	0.000	0.000	0.000	0.000	0.000
	Pseudo R2	0.0563	0.1104	0.122	0.0954	0.1605	0.1823	0.0708	0.0819	0.085	0.066	0.0827	0.0864
	Log pseudolikelihood	-75072753	-70759423	-68976022	-54457640	-50522762	-48505398	-59776971	-59066470	-58360376	-55449230	-54457519	-53850223

Note: **®**Reference category

† *p* < 0.001

*** *p* < 0.01

** *p* < 0.05 and

* *p* < 0.1

Source: Authors’ own calculations from Longitudinal Aging Study in India (LASI), Main Wave I, (2017–18)

Interaction terms between sex and marital status; sex and living arrangement; and sex and involvement in intra-household financial matters were successively included in three additional models 4–6, each controlling for the co-variates considered in model 3. The results of the regressions, presented in Table 7, showed that the effect of ‘currently married’ status (model 4) and living arrangement (model 5) was not significantly different for older men and women in case of subjective health outcomes. Involvement in financial matters had a highly statistically significant stronger effect for men in reversing poor subjective health status. In case of objective measures, being currently married, living in non-solitary arrangements, had a stronger effect for older men compared to women in determining poor grip strength, while it had a stronger effect for women in determining poor balance (model 6).

**Table 7:** Effect of Interaction between gender and select co-variates in determining health outcomes.

Dependent Variable Yes = 1, No = 0		RESTRICTED MOBILITY			FUNCTIONAL LIMITATION			LOW GRIP STRENGTH			POOR STATIC BALANCE		
Interactions		MODEL 4	MODEL 5	MODEL 6	MODEL 4	MODEL 5	MODEL 6	MODEL 4	MODEL 5	MODEL 6	MODEL 4	MODEL 5	MODEL 6
		Adjusted Odds Ratio			Adjusted Odds Ratio			Adjusted Odds Ratio			Adjusted Odds Ratio		
Sex * Marital Status	Male * currently married**®**												
	Female * currently not married	1.28***			1.37***			0.56†			1.62†		
	Female *currently married	1.08			0.97			0.56†			1.33***		
	Male * currently not married	0.98			1.00			1.03			1.11		
Sex * Living Arrangement	Male * living alone**®**												
	Female * not living alone		0.89			0.99			0.41†			2.26†	
	Female * living alone		1.18			0.76			0.54***			2.14***	
	Male * not living alone		0.77			0.87			0.73			1.68**	
Sex * Involvement in financial matters	Male * involved in financial matters**®**												
	Female * not involved in financial matters			2.08†			2.68†			0.76†			1.82†
	Female * involved in financial matters			1.33***			1.36**			0.64†			1.41***
	Male * not involved in financial matters			1.88†			2.48†			1.46†			1.34***
**Model Specifications**	Number of observations	30,938	30,938	30,938	30,900	30,900	30,900	27,888	27,888	27,888	26,642	26,642	26,642
	Wald statistic	1460.79	1462.07	1462.62	1570.52	1559.61	1553.37	733.89	735.14	730.29	711.09	713.15	709.46
	Prob > chi2	0.000	0.000	0.000	0.000	0.000	0.000	0.000	0.000	0.000	0	0	0
	Pseudo R2	0.1223	0.122	0.1222	0.1829	0.1825	0.1825	0.085	0.085	0.0853	0.0865	0.0867	0.0864
	Log pseudolikelihood	-68956834	-68975968	-68961230	-48464840	-48493750	-48493506	-58360185	-58360318	-58342257	-53846260	-53833898	-53849835

Note: **®**Reference category

† *p* < 0.001

*** *p* < 0.01

** *p* < 0.05 and

* *p* < 0.1

Models 4, 5 and 6 control for all the co-variates considered in Model 3 in Table 6.

Source: Authors’ own calculations from Longitudinal Aging Study in India (LASI), Main Wave I, (2017–18)

## Discussion

The present paper made an attempt to explain the gender differentials in subjective and objective health status in the later stages of life. The findings of the present paper confirm the existence of significant gender differentials in both subjective and objective health measures among the older Indian population. Except for low grip strength, older women tend to experience poorer health outcomes in terms of restricted mobility, functional limitation, and poor static balance compared to their male counterparts. In the case of low grip strength, older men were found at a more disadvantageous state of health. The gender differentials disfavouring women in functional limitation and poor static balance disappeared upon controlling financial empowerment in the model, while it still persisted in case of restricted mobility. The magnitude of gender difference disfavouring older men in case of low grip strength also shrunk but remained statistically significant. These findings, thus, support the presumption that there exist notable gender differences in the health status of the older adults put forward by some previous research [23, 24, 40].

The regression results showed that gender differentials persisted and disfavored women even after controlling for a range of demographic and social-support factors in the case of each health outcome except for grip strength. After adding socioeconomic factors and health conditions to the estimated model, the gender differential contracted but remained significant. However, upon controlling the effect of financial empowerment, gender differentials disfavouring women in functional limitation and poor static balance became statistically insignificant, thereby highlighting the role of financial empowerment in influencing health, especially among women in the later stage of life. Existing literature suggests that financial empowerment gives one economic stability which further helps reduce stress and live a healthy life [40, 78, 79]. Financial empowerment enables women to access primary and secondary prevention during the life-span, which helps to improve their health in later life. The present study also confirms the significant positive influence of financial empowerment in reducing gender differentials in health status.

In terms of other determinants of subjective and objective health status, marital status, social group, education, work status, economic status, place of residence, chronic ailment, and impairment were found to have a significant impact in the context of older Indian adults. Social group affiliation significantly impacts health, especially the objective health status of older adults, and this finding is in line with previous literature [48]. People belonging to the so-called lower castes often have to face obstacles in accessing resources related to health, nutrition, and education, making them more vulnerable to poor health status. Education is another crucial factor that determines the health status of older persons. On the one hand, it is the key to financial independence which in turn provides enough resources to take care of one’s health. On the other hand, it generates awareness which increases the adoption of preventive health care routine which ultimately provides better health status [80, 81]. Additionally, educated people have the choice of engaging themselves in jobs with less risk to health and more leisure time which may induce a sedentary life style.

Place of residence was also found to have a significant effect in explaining older adults’ health status. Older persons who reside in urban areas reported better subjective health status though the case is the opposite for objective health measures. This may reflect the fact that people in urban areas, having easier access to better health care services, report better health status though it may not be valid for measured health status [56]. Prevalence of chronic ailment and impairment also revealed a positive association of poor health status, especially for subjective or perceived health status. Older persons with chronic ailment and impairment reported poorer subjective and measured health outcomes compared to those with no chronic ailment and impairment. As chronic ailment and impairment can last for a long period of life-span, it drains people economically as well as mentally, which in turn hampers both perceived as well as measured health status.

Another crucial finding of this study is that though the effect of ‘currently married’ status was not significantly different for men and women in determining subjective health status, it was found to be significant in determining objective health status. A similar result emerged for the effect of living arrangement on the health of older men and women. This supports the evidence provided by earlier literature suggesting that widowed men have higher health disadvantage than married men, while in the case of women, the corresponding differential is smaller [47, 82]. The health advantage in terms of marital status also reflects the fact that in later life, friendship ties become weak, and most people get emotional support from their spouse. In addition, older women have a higher chance of widowhood because of the fact that women live longer than men, even with higher morbidity [83]. Besides, the high prevalence of the nuclear family system makes the widow/widower live alone and more isolated. Due to no support or lack of care giver, the health status of such an individual deteriorates further.

Intra-household decision-making was also found to be an important determinant of both objective and subjective health status in the Indian context. Evidence suggests that household decision-making is positively linked with health status [84]. When older adults are the decision-makers, they are more likely to receive better care leading to better health status [85]. On the other hand, there might be an opposite relationship between decision-making and health status as those with poor health status cannot actively participate in household decision-making, especially in financial matters [86]. However, in the Indian context, decision-making has been chiefly dominated by men as compared to women [87] and consequently, a decrease in the decision-making role in the household makes men more disadvantageous than women [88].

## Conclusion

To sum up, this study provided scientific evidence towards persisting gender differentials in subjective and objective health status, most of which can be explained by financial empowerment. The results suggest that financial empowerment enables a woman to take both preventive and curative health care from their early life which help them to retain better health status in their old age as well. Moreover, the health advantage of financial empowerment is not restricted to women. These findings suggest an important policy implication of encouraging financial empowerment irrespective of gender in developing countries such as India. In a resource-poor setting where both the absolute and relative population of older adults are continuously rising, financial empowerment can be a way to cover-up the supply-side constraints. To increase financial awareness, the government and other stakeholders should organize awareness camps. The coverage, outreach as well as benefits of programmes like the Indira Gandhi National Old Age Pension Scheme (IGNOAPS) [89] should be improved to ensure financial independence in the older ages which will help to improve their health status. Moreover, guaranteeing women’s property as well as inheritance rights, and providing equal access to educational and economic opportunities are important policy measures in the direction of reducing gender disparity.

The present study is constrained by the limitations of cross-sectional analysis that does not provide evidence for making inferences on cause and effect [90]. Also, the lack of data on the degree of difficulty faced in mobility and activities of daily living was a deterrent in constructing the self-reported (subjective) health outcomes more robustly. However, despite these limitations, the findings of the study have made an important contribution to the understanding of the determinants of gender differential in older adults’ health, in general, and the influence of financial empowerment on health outcomes, in particular, which may encourage researchers to study the topic further. Future studies may be conducted using longitudinal data once the data of successive waves of LASI are available for conclusive findings on the causal relationships.

## Supporting information

S1 Appendix Gender differentials in prevalence of poor subjective health outcomes by background characteristics. (DOCX)

S2 Appendix Gender differentials in prevalence of poor objective health outcomes by background characteristics. (DOCX)
