# Supplementary material for: Exploring the role of financial empowerment in mitigating the gender differentials in subjective and objective health outcomes among the older population in India
Source: PLoS One. 2023 Jan 23;18(1):e0280887. doi: 10.1371/journal.pone.0280887 (PMC9870167; doi:10.1371/journal.pone.0280887)
Supplement: S1 Appendix — (DOCX) [file pone.0280887.s001.docx]

# Exploring the role of financial empowerment in mitigating the gender differentials in subjective and objective health outcomes among the older population in India

# Supporting Information

**S1 Appendix. Gender differentials in prevalence of poor subjective health outcomes by background characteristics**

| Background Characteristics | | RESTRICTED MOBILITY | | | | | | | FUNCTIONAL LIMITATION | | | | | | |
| --- | --- | --- | --- | --- | --- | --- | --- | --- | --- | --- | --- | --- | --- | --- | --- |
|  |  | Total |  | Male |  | Female |  | Male-female difference | Total |  | Male |  | Female |  | Male-female difference |
|  |  | % |  | % |  | % |  |  | % |  | % |  | % |  |  |
| Age group | Younger olds (60-69 years) | 30.86 | † | 24.19 | † | 36.74 | † | -12.55 | 13.68 | † | 8.55 | † | 18.21 | † | -9.66 |
|  | Older Olds (70-79 years) | 48.61 |  | 39.97 |  | 56.67 |  | -16.70 | 24.38 |  | 17.09 |  | 31.18 |  | -14.09 |
|  | Oldest olds (80 years and above) | 61.88 |  | 56.87 |  | 66.19 |  | -9.32 | 46.44 |  | 39.33 |  | 52.54 |  | -13.21 |
| Marital Status | Currently Married | 34.11 | † | 31.01 | † | 39.2 | † | -8.19 | 15 | † | 13.03 | † | 18.23 | † | -5.2 |
|  | Widowed | 49.38 |  | 40.4 |  | 51.85 |  | -11.45 | 30.08 |  | 21.42 |  | 32.47 |  | -11.05 |
|  | Others | 35.75 |  | 35.18 |  | 36.31 |  | -1.13 | 21.59 |  | 21.25 |  | 21.92 |  | -0.67 |
| Social Group | SC | 39.58 | † | 32.88 | † | 45.54 | † | -12.66 | 22.95 | † | 16.77 | *** | 28.45 | † | -11.68 |
|  | ST | 33.92 |  | 29.02 |  | 37.93 |  | -8.91 | 18.42 |  | 12.28 |  | 23.43 |  | -11.15 |
|  | OBC | 39.67 |  | 33.6 |  | 45.19 |  | -11.59 | 21.17 |  | 15.35 |  | 26.46 |  | -11.11 |
|  | Others | 41.49 |  | 31.95 |  | 49.97 |  | -18.02 | 18.71 |  | 12.47 |  | 24.28 |  | -11.81 |
| Religion | Muslim | 43.68 | † | 35.4 | † | 51.2 | † | -15.80 | 21.52 | † | 14.67 | *** | 27.72 | † | -13.05 |
|  | Hindu | 39.31 |  | 32.28 |  | 45.61 |  | -13.33 | 20.71 |  | 14.51 |  | 26.27 |  | -11.76 |
|  | Others | 37.83 |  | 32.62 |  | 42.19 |  | -9.57 | 17.68 |  | 15.32 |  | 19.65 |  | -4.33 |
| Living Arrangement | Alone | 49.42 | † | 42 | † | 51.37 | † | -9.37 | 23.77 | † | 22.35 | *** | 24.14 | † | -1.79 |
|  | With children and spouse/ others | 39.75 |  | 31.79 |  | 46.81 |  | -15.02 | 21.14 |  | 14.06 |  | 27.42 |  | -13.36 |
|  | With spouse and/or others | 37.36 |  | 33.86 |  | 41.4 |  | -7.54 | 18.42 |  | 15.12 |  | 22.23 |  | -7.11 |
| Education | illiterate | 43.88 | † | 37.86 | † | 46.85 | † | -8.99 | 25.9 | † | 19.94 | † | 28.84 | † | -8.9 |
|  | upto primary | 38.34 |  | 34.2 |  | 45.32 |  | -11.12 | 15.24 |  | 13.52 |  | 18.15 |  | -4.63 |
|  | secondary | 30.4 |  | 25.58 |  | 41.17 |  | -15.59 | 12.14 |  | 8.76 |  | 19.7 |  | -10.94 |
|  | higher secondary or above | 26.23 |  | 22.59 |  | 39.88 |  | -17.29 | 7.89 |  | 7.98 |  | 7.55 |  | 0.43 |
| Work Status | Never worked | 46.01 | † | 40.16 | † | 46.44 | † | -6.28 | 25.47 | † | 24.89 | † | 25.51 | † | -0.62 |
|  | currently not working/ unpaid work | 46.49 |  | 41.89 |  | 52.79 |  | -10.90 | 26.4 |  | 20.97 |  | 33.84 |  | -12.87 |
|  | Currently working (paid) | 23.64 |  | 20.02 |  | 31.23 |  | -11.21 | 7.36 |  | 5.37 |  | 11.54 |  | -6.17 |
| Economic Status | Poorest | 41.29 |  | 34.14 |  | 47.25 |  | -13.11 | 24.75 | † | 17.9 | † | 30.47 | † | -12.57 |
|  | Poorer | 39.93 |  | 33.78 |  | 45.28 |  | -11.50 | 20.71 |  | 15.77 |  | 25 |  | -9.23 |
|  | Middle | 38.57 |  | 31.75 |  | 44.83 |  | -13.08 | 18.98 |  | 13.26 |  | 24.23 |  | -10.97 |
|  | Richer | 38.22 |  | 31.85 |  | 44 |  | -12.15 | 19.92 |  | 12.3 |  | 26.83 |  | -14.53 |
|  | Richest | 40.39 |  | 31.37 |  | 49.04 |  | -17.67 | 17.79 |  | 13.2 |  | 22.19 |  | -8.99 |
| Place of Residence | Rural | 40.51 | *** | 33.65 | † | 46.94 |  | -13.29 | 21.97 | † | 16 | † | 27.58 | † | -11.58 |
|  | Urban | 37.71 |  | 29.98 |  | 43.84 |  | -13.86 | 17.25 |  | 10.79 |  | 22.36 |  | -11.57 |
| Chronic disease | None | 29.05 | † | 22.82 | † | 35.04 | † | -12.22 | 15.84 | † | 10.24 | † | 21.23 | † | -10.99 |
|  | only one | 39.15 |  | 32.53 |  | 45.12 |  | -12.59 | 19.51 |  | 13.8 |  | 24.65 |  | -10.85 |
|  | two or more | 52.52 |  | 45.18 |  | 58.5 |  | -13.32 | 27.15 |  | 20.85 |  | 32.28 |  | -11.43 |
| Impairment | None | 37.45 | † | 29.82 | † | 44.24 | † | -14.42 | 18.29 | † | 12.29 | † | 23.64 | † | -11.35 |
|  | only one | 52.8 |  | 52.56 |  | 53.03 |  | -0.47 | 34.45 |  | 30.54 |  | 38.46 |  | -7.92 |
|  | two or more | 68.71 |  | 64.17 |  | 72.12 |  | -7.95 | 49.77 |  | 40.34 |  | 56.84 |  | -16.5 |
| Role in property related decisions | No role | 50.63 | † | 48 | † | 51.55 | † | -3.55 | 37.05 | † | 31.71 | † | 38.9 | † | -7.19 |
|  | Decide alone | 29.14 |  | 25.05 |  | 39.59 |  | -14.54 | 11.3 |  | 8.36 |  | 18.8 |  | -10.44 |
|  | Decides jointly | 38.51 |  | 32.28 |  | 44.55 |  | -12.27 | 17.53 |  | 13.59 |  | 21.36 |  | -7.77 |
| Involvement in payment of bills/ settling of financial matters | No | 46.95 | † | 44.17 | † | 48.37 | † | -4.20 | 26.76 | † | 23.75 | † | 28.3 | † | -4.55 |
|  | Yes | 24.47 |  | 21.76 |  | 33.02 |  | -11.26 | 7.26 |  | 5.85 |  | 11.71 |  | -5.86 |
| Financial support | Received and given | 37.76 | † | 31.76 | † | 45.42 | † | -13.66 | 19.47 | † | 11.49 | † | 29.67 | † | -18.18 |
|  | Received but not given | 44.55 |  | 37.34 |  | 49.75 |  | -12.41 | 25.24 |  | 19.44 |  | 29.43 |  | -9.99 |
|  | Not received but given | 29.54 |  | 25.99 |  | 36.88 |  | -10.89 | 11.67 |  | 7.9 |  | 19.48 |  | -11.58 |
|  | Neither received nor given | 39.62 |  | 32.54 |  | 45.89 |  | -13.35 | 20.55 |  | 14.43 |  | 25.39 |  | -10.96 |
| TOTAL |  | 39.69 |  | 32.65 |  | 45.99 | † | -13.34 | 20.6 |  | 14.58 |  | 25.98 | † | -11.4 |

Note: † *p* < 0.001, *** *p* < 0.01 ** *p* < 0.05 and * *p* < 0.1

Source: Authors’ own calculations from Longitudinal Ageing Study in India (LASI), Main Wave I, (2017-18)
