# Supplementary material for: Exploring the role of financial empowerment in mitigating the gender differentials in subjective and objective health outcomes among the older population in India
Source: PLoS One. 2023 Jan 23;18(1):e0280887. doi: 10.1371/journal.pone.0280887 (PMC9870167; doi:10.1371/journal.pone.0280887)
Supplement: S2 Appendix — (DOCX) [file pone.0280887.s002.docx]

# Exploring the role of financial empowerment in mitigating the gender differentials in subjective and objective health outcomes among the older population in India

# Supporting Information

**S2 Appendix. Gender differentials in prevalence of poor objective health outcomes by background characteristics**

| Background Characteristics | | LOW GRIP STRENGTH | | | | | | | POOR STATIC BALANCE | | | | | | | |
| --- | --- | --- | --- | --- | --- | --- | --- | --- | --- | --- | --- | --- | --- | --- | --- | --- |
|  |  | Total |  | Male |  | Female |  | Male-female difference | Total |  | Male |  | Female |  | Male-female difference |  |
|  |  | % |  | % |  | % |  |  | % |  | % |  | % |  |  |  |
| Age group | Younger olds (60-69 years) | 60.82 | † | 63.81 | † | 58.18 | † | 5.63 | 22.63 | † | 16.17 | † | 28.36 | † | -12.19 |  |
|  | Older Olds (70-79 years) | 81.71 |  | 82.8 |  | 80.68 |  | 2.12 | 31.73 |  | 25.85 |  | 37.57 |  | -11.72 |  |
|  | Oldest olds (80 years and above) | 91.13 |  | 91.02 |  | 91.23 |  | -0.21 | 51.49 |  | 47.09 |  | 55.72 |  | -8.63 |  |
| Marital Status | Currently Married | 67.42 | † | 70.68 | † | 62.11 | † | 8.57 | 23.14 | † | 20.51 | † | 27.4 | † | -6.89 |  |
|  | Widowed | 74.97 |  | 80.58 |  | 73.39 |  | 7.19 | 37.09 |  | 30.13 |  | 39.12 |  | -8.99 |  |
|  | Others | 69.51 |  | 78 |  | 60.68 |  | 17.32 | 28.31 |  | 24.43 |  | 32.38 |  | -7.95 |  |
| Social Group | SC | 72.67 | † | 75.8 | † | 69.8 | † | 6 | 25.97 | † | 22.22 | † | 29.41 | † | -7.19 |  |
|  | ST | 72.66 |  | 75.12 |  | 70.66 |  | 4.46 | 20.62 |  | 15.05 |  | 25.13 |  | -10.08 |  |
|  | OBC | 71.51 |  | 73.97 |  | 69.25 |  | 4.72 | 29.81 |  | 23.03 |  | 36.16 |  | -13.13 |  |
|  | Others | 65.4 |  | 66.82 |  | 64.1 |  | 2.72 | 28.89 |  | 22.59 |  | 34.83 |  | -12.24 |  |
| Religion | Muslim | 68.68 | † | 70.29 | † | 67.16 | † | 3.13 | 27.44 | *** | 18.47 | ** | 36.01 | † | -17.54 |  |
|  | Hindu | 70.31 |  | 72.6 |  | 68.23 |  | 4.37 | 27.64 |  | 22.03 |  | 32.81 |  | -10.78 |  |
|  | Others | 70.61 |  | 74.21 |  | 67.42 |  | 6.79 | 34.72 |  | 30.16 |  | 38.71 |  | -8.55 |  |
| Living Arrangement | Alone | 77.09 | † | 81.26 | † | 76.01 | † | 5.25 | 32.72 | † | 20.64 | *** | 35.95 | ** | -15.31 |  |
|  | With children and spouse/ others | 68.91 |  | 71.12 |  | 66.91 |  | 4.21 | 27.57 |  | 21.37 |  | 33.35 |  | -11.98 |  |
|  | With spouse and/or others | 72 |  | 74.92 |  | 68.6 |  | 6.32 | 28.41 |  | 24.22 |  | 33.23 |  | -9.01 |  |
| Education | illiterate | 73.37 | † | 80.01 | † | 70.06 | † | 9.95 | 29.75 | † | 23.54 | † | 32.85 |  | -9.31 |  |
|  | upto primary | 69.39 |  | 71.75 |  | 65.23 |  | 6.52 | 26.86 |  | 22.61 |  | 34.48 |  | -11.87 |  |
|  | secondary | 63.13 |  | 65.39 |  | 58.12 |  | 7.27 | 25.94 |  | 19.81 |  | 41.19 |  | -21.38 |  |
|  | higher secondary or above | 58.76 |  | 59.11 |  | 57.38 |  | 1.73 | 21.46 |  | 20.18 |  | 26.79 |  | -6.61 |  |
| Work Status | Never worked | 66.53 | † | 74.05 | † | 65.98 | † | 8.07 | 36.01 | † | 27.98 | † | 36.59 | † | -8.61 |  |
|  | Currently not working/ unpaid work | 76.66 |  | 78.29 |  | 74.4 |  | 3.89 | 32.23 |  | 29.25 |  | 36.35 |  | -7.10 |  |
|  | Currently working (paid) | 63.83 |  | 65.03 |  | 61.29 |  | 3.74 | 15.73 |  | 13.18 |  | 21.13 |  | -7.95 |  |
| Economic Status | Poorest | 73.16 | † | 76.87 | † | 70.03 | † | 6.84 | 25.42 | † | 21.61 | ** | 28.66 | † | -7.05 |  |
|  | Poorer | 71.28 |  | 75.05 |  | 67.85 |  | 7.2 | 26.81 |  | 21.64 |  | 31.49 |  | -9.85 |  |
|  | Middle | 69.64 |  | 70.08 |  | 69.22 |  | 0.86 | 28.77 |  | 24.65 |  | 32.64 |  | -7.99 |  |
|  | Richer | 68.59 |  | 71.56 |  | 65.86 |  | 5.7 | 29.96 |  | 20.17 |  | 39.11 |  | -18.94 |  |
|  | Richest | 67.19 |  | 67.61 |  | 66.79 |  | 0.82 | 30.18 |  | 22.63 |  | 37.87 |  | -15.24 |  |
| Place of Residence | Rural | 70.71 | † | 73.24 | † | 68.29 | ** | 4.95 | 26.78 | † | 21.56 | † | 31.78 | † | -10.22 |  |
|  | Urban | 68.74 |  | 70.24 |  | 67.55 |  | 2.69 | 31.45 |  | 23.8 |  | 37.87 |  | -14.07 |  |
| Chronic disease | None | 70.29 | *** | 71.88 | *** | 68.73 | * | 3.15 | 23.85 | † | 18.73 | † | 28.88 | † | -10.15 |  |
|  | only one | 70.46 |  | 72.41 |  | 68.66 |  | 3.75 | 26.97 |  | 22.74 |  | 30.93 |  | -8.19 |  |
|  | two or more | 69.68 |  | 73.23 |  | 66.79 |  | 6.44 | 34.35 |  | 26.04 |  | 41.34 |  | -15.30 |  |
| Impairment | None | 69.44 | † | 71.68 | † | 67.42 | † | 4.26 | 27.22 | † | 21.28 | † | 32.66 | † | -11.38 |  |
|  | only one | 77.61 |  | 80.66 |  | 74.3 |  | 6.36 | 37.54 |  | 30.38 |  | 45.5 |  | -15.12 |  |
|  | two or more | 76.37 |  | 77.91 |  | 75.11 |  | 2.8 | 36.45 |  | 32.09 |  | 40.11 |  | -8.02 |  |
| Role in property related decisions | No role | 76.57 | † | 81.59 | † | 74.92 | † | 6.67 | 39.36 | † | 27.37 | † | 43.2 | † | -15.83 |  |
|  | Decide alone | 69.16 |  | 69.69 |  | 67.74 |  | 1.95 | 23.61 |  | 20.58 |  | 31.74 |  | -11.16 |  |
|  | Decides jointly | 68.82 |  | 71.91 |  | 65.8 |  | 6.11 | 26.05 |  | 21.85 |  | 30.24 |  | -8.39 |  |
| Involvement in payment of bills/ settling of financial matters | No | 73.22 | † | 80.45 | † | 69.51 | † | 10.94 | 32.74 | † | 28.1 | † | 35.12 | † | -7.02 |  |
|  | Yes | 64.01 |  | 64.98 |  | 60.94 |  | 4.04 | 18.87 |  | 16.81 |  | 25.4 |  | -8.59 |  |
| Financial support | Received and given | 72.5 | † | 75.95 | † | 67.95 | ** | 8 | 22.6 | † | 22.06 | † | 23.33 |  | -1.27 |  |
|  | Received but not given | 72.42 |  | 76.73 |  | 69.27 |  | 7.46 | 29.98 |  | 25.7 |  | 33.2 |  | -7.50 |  |
|  | Not received but given | 66.15 |  | 65.43 |  | 67.67 |  | -2.24 | 21.37 |  | 16.23 |  | 32.42 |  | -16.19 |  |
|  | Neither received nor given | 70.03 |  | 72.24 |  | 68.06 |  | 4.18 | 28.27 |  | 22.05 |  | 33.92 |  | -11.87 |  |
| TOTAL |  | 70.15 |  | 72.45 |  | 68.06 | † | 4.39 | 28.08 |  | 22.15 |  | 33.55 | † | -11.40 |  |

Note: † *p* < 0.001, *** *p* < 0.01 ** *p* < 0.05 and * *p* < 0.1

Source: Authors’ own calculations from Longitudinal Ageing Study in India (LASI), Main Wave I, (2017-18)
